# The Effect of Endotoxin on the Intestinal Mucus Layer in Non- and Post-pregnancy Mice

**DOI:** 10.3389/fvets.2021.824170

**Published:** 2022-02-10

**Authors:** Dujian Yan, Yuyun Qiang, Tian Tian, Dezhang Lu, Chenchen Wu

**Affiliations:** ^1^College of Veterinary Medicine, Northwest A&F University, Yangling, China; ^2^AKS Vocational and Technical College, Aksu, China

**Keywords:** lipopolysaccharide, MUC2, intestines, uterus, fetus, mucosal immunity

## Abstract

The intestine is the most extensive storage organ of bacteria and endotoxins, and the mucosal immune system is the first barrier of the intestine. Mucin-2 (MUC2) is the major component of the mucus layers. In this study, we explored whether MUC2 plays a role in how lipopolysaccharide (LPS) invades the fetus from the gut to the uterus in pregnant mice. The results showed that the LPS levels of the ileum, colon, and uterus were significantly increased, and the content of secretory IgA (sIgA) in the ileum, colon, and uterus tissues was significantly decreased in the LPS(+) group on the 35th day after LPS treatment. On the 16th day of pregnancy, compared with the LPS(-) group, the level of ileum LPS was significantly decreased, and the content of LPS in the fetus was significantly increased in the LPS(+) group. The sIgA content in the fetus was significantly decreased in the uterus and placenta. The expression of MUC2 in the uterus, ileum, and colon was increased significantly in the LPS(+) group, especially in the uterus. It is suggested that endotoxins accumulate in the uterus during non-pregnancy. The high expression of MUC2 in the uterus can prevent LPS from translocating into uterine tissue. After pregnancy, MUC2 still protects uterine tissue, allowing a large amount of LPS to enter the fetal body through blood circulation. Therefore, the level of sIgA significantly decreased, resulting in a decline in fetal innate immune function.

## Introduction

Endotoxin (lipopolysaccharide), a poisonous component in the outer membrane of most gram-negative bacteria (1, 2), is released in large quantities after the death of bacteria. Endotoxin is stable and exists widely in the environment, and it can cause endotoxemia by entering the body through the respiratory tract, digestive tract, and reproductive tract. The mucosal immune system (MIS) is the first immune barrier against infection and includes the mucosal tissue of the gastrointestinal, respiratory and genitourinary tracts, lymphoid tissue, and immunoreactive cells in the exocrine glands. The mucus layer was stimulated directly by external antigens (e.g., food, commensal bacteria, harmful pathogens). It can fight off the invasion of harmful substances (3, 4). Mucin-2 (MUC2) is the main component of the mucus layer and is secreted by goblet cells (5). The O-type oligosaccharide chain structure of MUC-2 provides binding sites for secretory IgA (sIgA) and antimicrobial peptides (6). MUC2 can be absorbed by dendritic cells after bacteria or endotoxins contact epithelial cells, reducing the production of proinflammatory factors dependent on carbohydrates (7). Previous studies have shown that endotoxin has long-term effects in the intestinal mucus layer. There was a significant increase in the secretion of MUC2 from intestinal goblet cells to enhance the defense function of the mucus layer. The goblet cells were subsequently released into the intestinal lumen in the form of compound exocytosis, the intestinal mucus layer became thinner, and the flora migrated to the intestinal epithelium (8). LPS in the internal environment of the reproductive organs is mainly derived from a bacterial infection of the reproductive tract and a translocation of lumen endotoxin. LPS affects the development of embryos, such as embryo dissolution, fetal death, and spontaneous abortion (9). How does intestinal LPS invade the fetus from the gut to the uterus in pregnant mice? Furthermore, how does it affect MUC2 expression in the intestine and uterus in the experimental period? Mature and pregnant female mice were administered LPS. We observed histomorphology changes in the tissues of the intestine, uterus, placenta, and fetus. We analyzed the content of sIgA in the blood, intestine, uterus, placenta, and fetus to provide a theoretical and scientific basis for the defense of the MIS against foreign toxic substances.

## Materials and Methods

### Mice

**Figure 1:**
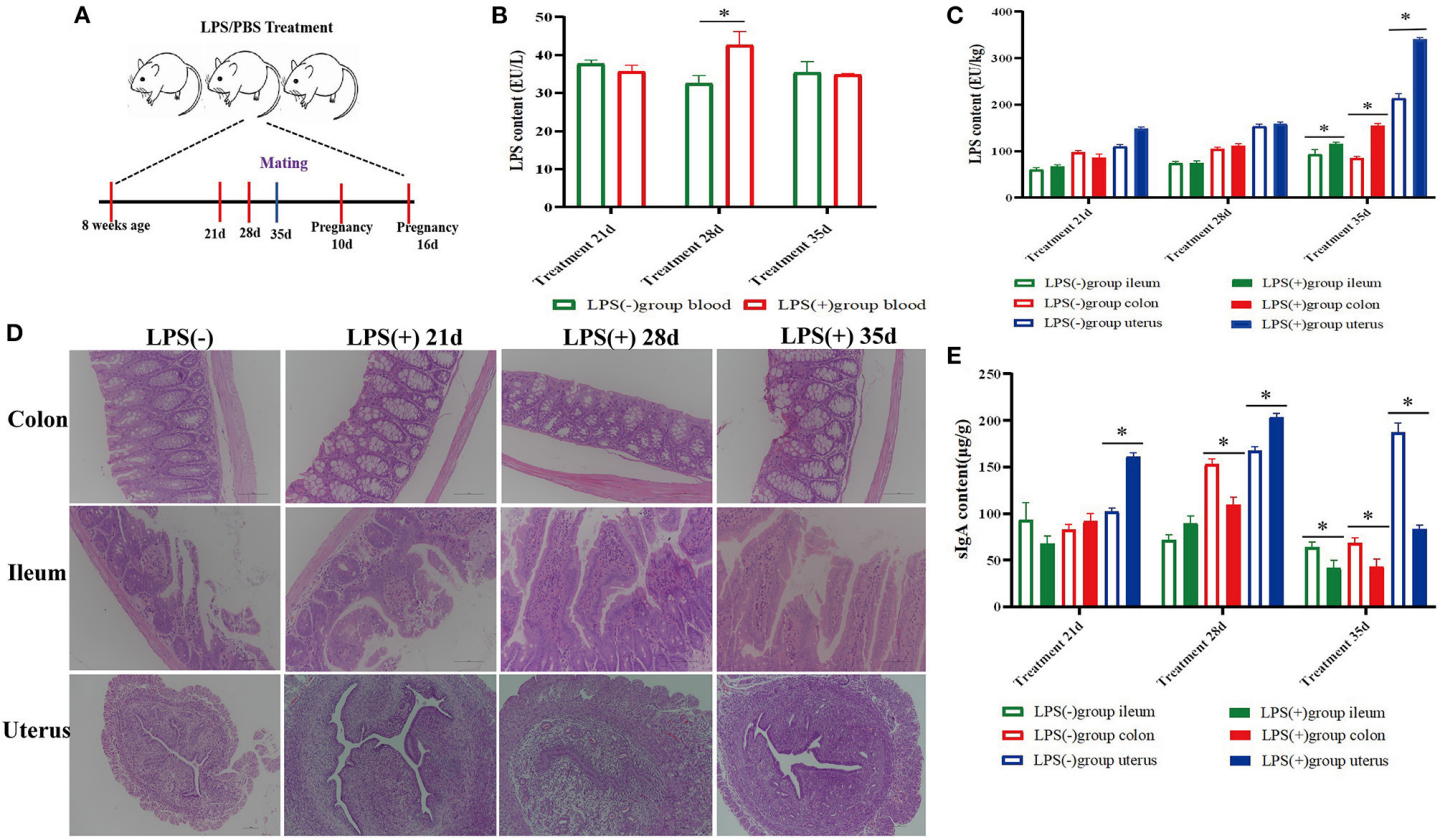
Effect of mouse LPS treatment for 35 days on histomorphology and biochemical indices of ileum, colon, and uterus tissues. **(A)** Experimental design; **(B)** LPS content in the blood of mice treated with LPS for 21 days (*n* = 10), 28 days (*n* = 10), and 35 days (*n* = 10). **(C)** LPS content in the colon, ileum, and uterine tissue of mice treated with LPS for 21 days (*n* = 10), 28 days (*n* = 10), and 35 days (*n* = 10). **(D)** Histomorphological changes in the colon, ileum, and uterine tissue of mice treated with LPS for 21 days (*n* = 10), 28 days (*n* = 10), and 35 days (*n* = 10). **(E)** SIgA content in the colon, ileum, and uterus of mice treated with LPS for 21 days (*n* = 10), 28 days (*n* = 10), and 35 days (*n* = 10). *p <0.05 indicates a significant difference between the LPS(+) group and the LPS(−) group during the same period.

The animal center performed all animal experiments according to the Guide for the Care and Use of Laboratory Animals using protocols approved by the Institutional Animal Care and Use Committee at Northwest A&F University (9). A total of 100 female mice (C57BL/6, 6 weeks old) were randomly divided into two groups: the LPS(+) group was orally gavaged with LPS (1 mg/kg; lipopolysaccharides from *Escherichia coli* O55:B5; Sigma, St. Louis, MO, USA), and the LPS(-) group was gavaged with 0.01 M phosphate buffer saline (PBS). After 35 days of LPS/PBS treatment, all of the female mice and normal male mice were coupled in cages. The day of pregnancy was recorded as 0 day when vaginal suppository was found in female mice. During the whole experimental period, all female mice were administered LPS/PBS by intragastric administration every other day, and their intake of chow and water was autoclaved. They were raised indoors at 26 ± 1°C with a relative humidity of ~50% (Figure 1A).

### Sample Collection

Blood, amniotic fluid, ileum, colon, uterus, placenta, and fetus were collected at 21, 28, and 35 days and at 10 and 16 days of pregnancy after LPS/PBS treatment. The plasma (10% heparin sodium) and amniotic fluid were centrifuged at 3,000 rpm for 10 min, and then supernatants were stored at −20°C. Parts of the ileum, colon, uterus, placenta, and fetus samples were fixed in 10% formalin for morphological analysis, and other parts were weighed and homogenized by hand (1 g of tissue in 10 ml of 0.01 M PBS), centrifuged for 20 min at 2,500 rpm, and the supernatants were stored at −20°C.

### Histomorphology

After fixation, the tissues were pruned, dehydrated with alcohol at different concentrations, and embedded in paraffin wax. Tissue sections (5 μm) were cut with a microtome, expanded in water at 43°C, and then mounted on slides. All of the sections were dried at 60°C. Morphological changes in the ileum, colon, and uterus were observed by hematoxylin and eosin (H&E) staining, consisting of xylene dewaxing → hydration → H&E staining → dehydration → sealing. The sections were placed under a microscope to observe the pathological and histological changes in the mouse ileum, colon, uterus, placenta, and fetus.

### Biochemical Indicators

The ileum, colon, uterus, placenta, and fetus of female mice were centrifuged, and supernatants were collected. The levels of LPS, sIgA, IgG, C1GalT1 (core 1β-1,3-galactosyltransferase), C2GnTs (core 2β-1,6-N-acetylglusaminyltransferase), and C3GnT (core 3β-1,3-N-acetylglucosaminyltransferase) in the tissue supernatants were determined by enzyme-linked immuno sorbent assay (ELISA) (Jingmei Biotechnology, Shanghai, China). According to the kit instructions, 50 μl of standard was added to the standard well. A sample dilution of 40 μl was added to the testing sample well, and then 10 μl of the testing sample was added. After adding enzyme, the plate was closed and incubated for 60 min at 37°C. Then, the liquid was discarded, and the remaining water was absorbed with bibulous paper. Following complete washing, Tetramethyl benzidine (TMB) substrate solution was added. The absorbances were measured at a 450 nm wavelength with an enzyme microplate reader by an enzyme standard instrument (HBS-1101). Assay range: 0.5–16 ng/ml. Sensitivity: The minimum detectable dose is typically < 0.1 ng/ml.

### Immunofluorescent Staining

Confocal microscopy was used to assess the localization of MUC2 (cat. no. ab90007; 5 μg; Abcam) in the ileum, colon, and uterus on Days 10 and 16 of pregnancy after LPS/PBS treatment, respectively. All samples were fixed with 4% paraformaldehyde for 10 min at 4°C and then washed three times with PBS. The samples were permeabilized with 0.1% Triton X-100 in PBS for 10 min. The ileum, colon, and uterus were incubated in blocking buffer for 3 h and then washed with Tris-Buffered Saline and Tween (TBST) three times. The tissues were incubated with an anti-MUC2 antibody. All antibodies were diluted in primary antibody dilution buffer for 2 h at 4°C and then washed three times. Subsequently, the tissues were incubated with Cy5-PEG-Biotin mouse anti-rabbit IgG (Bioss Antibodies, Woburn, MA, USA), diluted in secondary antibody dilution buffer for 2 h at 37°C in the dark and then stained with 2-(4-Amidinophenyl)-6-indolecarbamidine dihydrochloride (DAPI) for 5 min. Finally, the tissues were observed using an LSM780 immunofluorescence microscope (Carl Zeiss, Inc., Thornwood, NY, USA). All experiments were repeated at least three times.

### Statistical Analysis

The statistical analyses were accomplished using GraphPad Prism 7, and the data are expressed as the mean ± SD. The statistical analyses used the Statistical Package for Social Sciences (SPSS, USA) software, including *T*-test sample analysis. Each group vs. the control group was analyzed at the same time. ^*^P < 0.05 indicates a significant difference between the LPS(+) group and the LPS(-) group during the same period; ^**^P < 0.01 indicates an extremely significant difference between the LPS(+) group and the LPS(-) group during the same period.

## Results

### Effect of LPS on Histomorphology and Biochemical Indices in Non-pregnant Mice

The results showed that the colon and ileum tissues had different degrees of damage with prolonged LPS treatment time, especially in the ileum. Compared to the LPS(-) group, the ileum epithelial cells were destroyed; the intestinal villi were shortened, dissolved, and destroyed; and the intestinal lumen contained exfoliated intestinal villi and necrotic cells in the LPS(+) group mice 35 days after LPS treatment. However, there was an increase in the number of goblet cells among intestinal epithelial cells of the colon, and there was massive inflammatory cell infiltration in the lamina propria of the ileum and colon. No significant pathological changes were observed in the plasma membrane, endometrium, lamina propria, or uterine epithelium in the LPS(+) group. However, the number of lymphocytes and neutrophils increased in the lamina propria of the uterus in the LPS(+) group after LPS treatment on Day 35 (Figure 1D).

The level of LPS from blood in the LPS(+) group was significantly (*P* < 0.05) higher than that in the LPS(-) group at Day 28 of LPS treatment, and compared with the LPS(-) group, the changes in the content of LPS from blood were not significant (*P* > 0.05) in other experimental periods (Figure 1B). At the same time, the content of LPS in ileum, colon, and uterus tissues was significantly increased (*P* < 0.05) in comparison with the LPS(-) group at Day 35, and compared with the LPS(-) group, the changes in the content of LPS from the ileum, colon, and uterus tissues were not significant (*P* > 0.05) at Days 21 and 28 of LPS treatment (Figure 1C). Compared to the LPS(-) group, the level of sIgA in uterine tissues of mice was significantly increased (*P* < 0.05) at Days 21 and 28 of LPS treatment; however, the level of sIgA in colon tissues was significantly decreased (*P* < 0.05) at Day 28 of LPS treatment, and the level of sIgA in the ileum, colon, and uterine tissues was significantly decreased (*P* < 0.05) at Day 35 of LPS treatment (Figure 1E).

### Effect of LPS on Histomorphology and Biochemical Indices in Pregnant Mice

**Figure 2:**
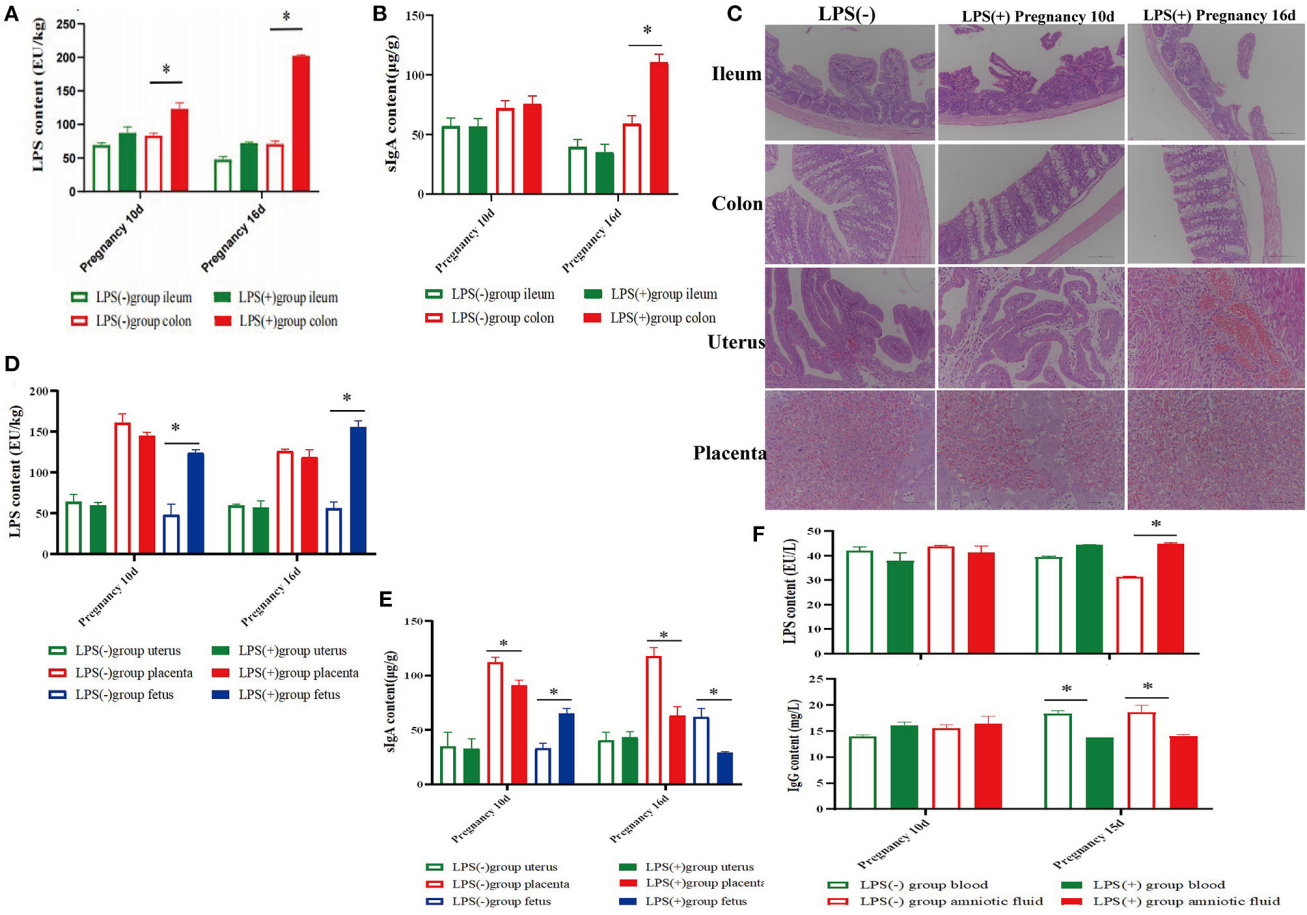
Effect of LPS on histomorphology and biochemical indices during pregnancy in mice. **(A)** Content of LPS in ileum and colon tissues of mice at 10 and 16 days of pregnancy. **(B)** Content of SIgA in ileum and colon tissues of mice at 10 and 16 days of pregnancy. **(C)** Histomorphological changes in the ileum, colon, uterus, and placenta of mice at 10 and 16 days of pregnancy. **(D)** Content of LPS in the uterus, placenta, and fetus of mice at 10 and 16 days of pregnancy. **(E)** Content of SIgA in the uterus, placenta, and fetus of mice at 10 and 16 days of pregnancy. **(F)** Contents of LPS and IgG in the blood and amniotic fluid of mice at 10 and 16 days of pregnancy. *p < 0.05 indicates a significant difference between the LPS(+) group and the LPS(−) group during the same period.

On the 10th and 16th day after pregnancy following treatment with LPS, the LPS level in the colon of the LPS(+) group was significantly decreased compared to LPS(-) group mice (*P* < 0.05), and the LPS level in the ileum of the LPS(+) group was lower than that of the LPS(-) group mice (*P* > 0.05; Figure 2A). Moreover, the content of sIgA in colon tissues of the LPS(+) group was significantly increased (*P* < 0.05) at Day 16 of pregnancy compared to that in the LPS(-) group (Figure 2B). On the 16th day after pregnancy, compared to the LPS(-) group, the changes in the content of LPS from uterine and placental tissues were not significant (P>0.05), but the content of LPS from fetuses in the LPS(+) group was significantly increased (*P* < 0.05; Figure 2D). On the 10th and 16th days after pregnancy, compared with the LPS(-) group, the content of sIgA in uterine tissues showed no significant changes (*P* > 0.05), and the level of sIgA in placental tissues was significantly decreased (*P* < 0.05) in the LPS(+) group. At the same time, the sIgA content from the fetus was significantly increased at Day 10 of pregnancy (*P* < 0.05), but the sIgA content from the fetus in the LPS(+) group was significantly decreased at Day 16 of pregnancy following treatment with LPS (*P* < 0.05; Figure 2E). As shown in Figure 2C, the histomorphology of the ileum and colon of the pregnant mice in the LPS(+) group was not significantly different from that of the LPS(-) group on the 16th day after pregnancy, and intestinal villi of the ileum and colon had noticeable pathological changes. However, the placenta and endometrial interstitium of pregnant mice in the LPS(+) group showed significant hemorrhage and congestion at Days 10 and 16 of pregnancy. Compared with the LPS(-) group, the content of LPS from amniotic fluid was significantly increased in LPS(+) group mice on the 10th day after pregnancy following treatment with LPS (*P* < 0.05); there were no significant differences between the LPS(-) group mice and LPS(+) group mice on the 10th day after pregnancy (*P* > 0.05), and the level of IgG from blood in the LPS(+) group was significantly increased on Day 10 with LPS treatment (*P* < 0.05), but the level of IgG from blood and amniotic fluid was significantly decreased on Day 16 of pregnancy (*P* < 0.05; Figure 2F).

### Effect of LPS on the Expression of MUC2 and Glycosyltransferase Activity in Mouse Tissues

**Figure 3:**
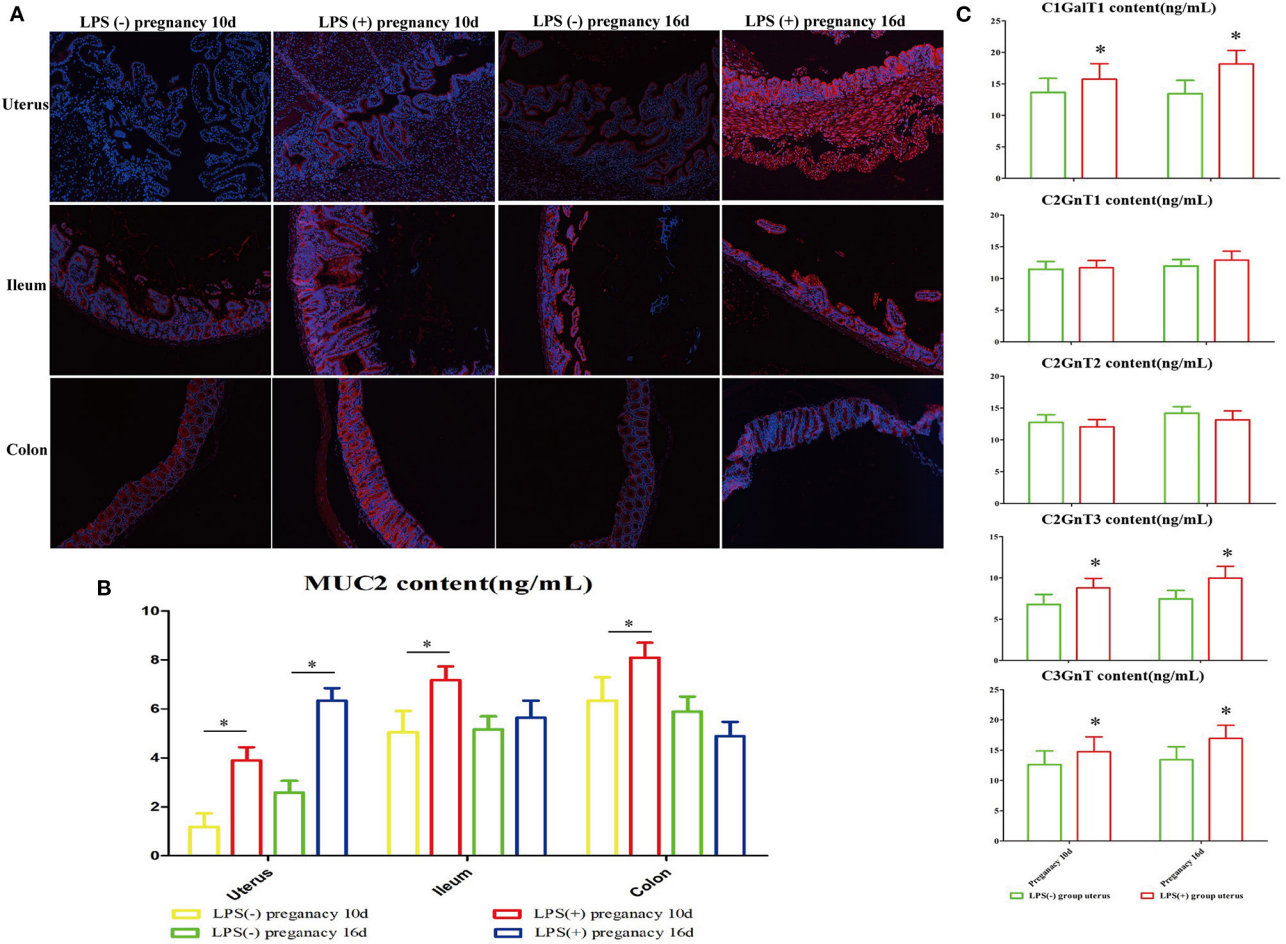
The effect of LPS on the expression of MUC2 in the intestinal and reproductive system mucosa. **(A)** Immunofluorescence staining was used to detect the expression of MUC2 in the ileum, colon, and uterus. **(B)** Changes in MUC2 content in the ileum, colon, and uterus. **(C)** The effect of LPS on mouse glycosyltransferase activity in the uterus (C1GalT1, C2GnT1, C2GnT2, C2GnT3, C3GnT). *p < 0.05 indicates a significant difference between the LPS(+) group and the LPS(−) group during the same period.

Immunofluorescent staining results showed that in comparison to the LPS(-) group, the expression of MUC2 in uterine, ileum, and colon tissues in the LPS(+) group increased significantly (*P* < 0.05) on Day 10 of pregnancy, and the expression of MUC2 in uterine tissues in the LPS(+) group increased significantly (*P* < 0.05) on Day 16 of pregnancy. However, the changes in the expression of MUC2 in ileum and colon tissues were not significant on Day 16 of pregnancy (Figures 3A,B). Furthermore, the analysis of uterine glycosyltransferase activity in mice at Days 10 and 16 of pregnancy indicated that the activities of C1GalT, C2GnT1, and C2GnT2 in the LPS(+) group were not significant on Days 10 and 16 of pregnancy compared to the LPS(-) group (*P* > 0.05). However, compared to the LPS(-) group, the activity of C2GnT3 in mouse uteri in the LPS(+) group was significantly increased at Days 10 and 16 of pregnancy (*P* < 0.05), and the activity of C3GnT in the LPS(+) group of mice was significantly decreased on Day 16 of pregnancy (*P* < 0.05; Figure 3C).

## Discussion

Lipopolysaccharide is a significant component of the outer membrane of gram-negative bacteria (10–12), and it is challenging to process lipopolysaccharide into toxoids due to its high stability and heat resistance. The intestine, the main digestive organ, is the largest storage organ of bacteria (13–15), which digests and absorbs various nutrients and prevents bacteria and their metabolites from translocating through the mucosal immune barrier (16). This research found that in comparison to the LPS(-) group, the LPS level of the ileum, colon, and cecum was significantly increased in the LPS(+) group on the 35th day after administering LPS by gavage. The LPS level of the uterus was higher than that of other tissues. Histomorphological damage to the ileum and colon was aggravated with prolonged LPS treatment. There were no obvious pathological changes in the histomorphology of the uterus, and the uterus had only a few infiltrated inflammatory cells. Therefore, we hypothesized that there must be large amounts of LPS adhered to endometrial epithelial cells that prevented them from entering the tissue on the 35th day after LPS treatment, which resulted in lower uterine damage than expected. Subsequently, female mice mated with healthy male mice on the 35th day after LPS treatment. On the 10th and 16th day of pregnancy, in comparison to the LPS(-) group, the level of colonic LPS in the LPS(+) group was significantly decreased. There were no significant morphological changes in the ileum and colon of pregnant mice, and the uterus and placenta appeared to be significantly congested and hemorrhagic. On the 16th day after pregnancy, compared with the LPS(-) group, the changes in the LPS content in the uterus and placenta were not significant. The above-mentioned results indicated that the intestinal damage induced by LPS in the non-pregnant period could be recovered through pregnancy. Therefore, we suspected that intestinal LPS entered the uterus and fetus *via* the blood circulation.

Compared with the LPS(-) group, the expression of MUC2 was significantly increased in the ileum, colon, and uterus of female mice in the LPS(+) group on the 10th day after pregnancy. On the 16th day after pregnancy, the expression of uterine MUC2 was significantly increased, and the changes in the expression of ileal and colonic MUC2 were not significant in the LPS(+) group. The first line of host defense against the encroachment of commensal bacteria and the invasion of enteric pathogens is the mucosal barrier, which is composed of mostly mucin glycoproteins (17). Mucus plays a critical role in reproductive function and defends the female reproductive tract (18). Mucus is synthesized and secreted by goblet cells, and its functions are primarily related to lubricating the epithelium and defending the epithelium against damaged substances (5). The mucus layer is the first line of defense against external antigens (5).

Mucus is a reservoir for other proteins, including sIgA and Adenosine monophosphate (AMPs) (17). In comparison with the LPS(-) group, the content of sIgA in colon tissues was significantly decreased; that in uterine tissues was significantly increased on the 28th day after LPS treatment, and the content of sIgA in ileum, colon, and uterine tissues was significantly lower in the LPS(+) group on the 35th day after LPS treatment. sIgA, the main factor of mucosal immunity, comprises the first line of antigen-specific immune defense, which prevents access to commensal and pathogenic microorganisms and their secreted products from entering the body (19). sIgA can bind antigen of the mucosa lamina propria and defend mucosal surfaces with an immune barrier against microbial adhesion. In addition, sIgA exerts anti-inflammatory, regulatory and modulatory functions (20). The results indicated that the LPS content in the ileum, colon and uterus tissues of mice in the LPS(+) group increased on the 35th day after LPS treatment. LPS invaded the uterus through the intestine and accumulated in large quantities in the uterus after oral gavage. On the 28th day after LPS treatment, the expression of uterine sIgA was increased due to the accumulation of LPS in the uterus for innate immunity. However, on the 35th day after LPS treatment, the decreased immunoglobulin sIgA expression in ileal, colonic, uterine, and immune cell necrosis indicated that LPS could cause immunosuppression.

Mucins are heavily glycosylated (40–80%) (21, 22), and O-glycosphere chains make up 50–80% of MUC2, which is closely associated with the function of mucin. The maturation of MUC2 is modified by o-linked glycosylation catalyzed with glycosyl transferases (23, 24). Compared to the LPS(-) group, the enzyme activities of C1GalT1, C2GnT3, and C3GnT were significantly increased in the LPS(+) group. These results suggested that LPS could activate the glycosylase of the MUC2-type O-glycan synthesis procedure and stimulate compensatory growth in goblet cells. In the upper mucus layer of the intestine, MUC2 creates a functional barrier between the host and luminal bacterial microbiota (25). MUC2 can envelope pathogenic bacteria, endotoxins, and other harmful substances on the surface of the intestinal mucosa. MUC2 is then taken up by intestinal dendritic cells to protect the intestinal mucosa and provide binding immune sites for sIgA and antimicrobial peptides (26). In comparison with the LPS(-) group, the sIgA content in the placenta and fetus was significantly decreased in the LPS(+) group on the 10th and 16th day after pregnancy, the content of IgG in blood was significantly increased in the LPS(+) group on the 10th day after pregnancy, and the content of IgG in blood was significantly decreased in the LPS(+) group on the 16th day after pregnancy. SIgA is the main immunoglobulin component of breast milk. In this study, the decreased sIgA levels in fetal and placental tissues indicated that LPS enters the fetus through the placental barrier with cord blood, which resulted in low levels of fetal antibodies and reduced immunity. IgG is the only immunoglobulin that can be transmitted to the fetus through the placenta. Small amounts of IgM and IgG exist in breast milk, and large amounts of immunoglobulins sIgA and IgM exist in transitional milk; furthermore, large amounts of IgG exist in mature milk (27). Maternal sIgA in milk has the greatest protective effect on neonates (27). The significant reduction in fetal sIgA content and maternal blood IgG LPS levels will increase the prevalence of infection and incidence of disease in newborn mice.

## Conclusion

In conclusion, the intestinal damage induced by LPS in the non-pregnant period could recover in the pregnant period. LPS entered the fetus through the placental barrier with blood, which resulted in low levels of fetal antibodies and reduced immunity. LPS could activate the glycosylase of the MUC2-type O-glycan synthesis process and stimulate compensatory growth in goblet cells. The high expression of MUC2 in the uterus might have a protective effect on the uterus; however, the protective function does not work if endometrial MUC2 expression is significantly increased. Mucus plays an important role in reproductive function and protects the uterus. The significant reductions in fetal sIgA content and maternal blood IgG LPS levels contribute to a low level of fetal autoimmunity.

## Data Availability Statement

The datasets presented in this study can be found in online repositories. The names of the repository/repositories and accession number(s) can be found in the article/supplementary material.

## Ethics Statement

The animal study was reviewed and approved by the Institutional Animal Care and Use Committee at Northwest A&F University.

## Author Contributions

CW, YQ, and DY: conceived and designed the experiments. YQ and TT: performed the experiments, supervision, and writing—original draft. YQ, TT, and CW: analyzed the data. DL, TT, DY, and CW: methodology. DL and CW: writing—review and editing. All authors contributed to the article and approved the submitted version.

## Funding

This study was supported by the Shaanxi province key research project (Grant No. 2020 NY-020).

## Conflict of Interest

The authors declare that the research was conducted in the absence of any commercial or financial relationships that could be construed as a potential conflict of interest.

## Publisher's Note

All claims expressed in this article are solely those of the authors and do not necessarily represent those of their affiliated organizations, or those of the publisher, the editors and the reviewers. Any product that may be evaluated in this article, or claim that may be made by its manufacturer, is not guaranteed or endorsed by the publisher.
